# Association of Lifelines Diet Score (LLDS) and metabolically unhealthy overweight/obesity phenotypes in women: a cross-sectional study

**DOI:** 10.1186/s12905-022-01957-x

**Published:** 2022-09-12

**Authors:** Alireza Khadem, Farideh Shiraseb, Atieh Mirzababaei, Rasool Ghaffarian-Ensaf, Khadijeh Mirzaei

**Affiliations:** 1 Department of Nutrition, Science and Research Branch, Islamic Azad University, Tehran, Iran; 2 Department of Community Nutrition, School of Nutritional Sciences and Dietetics, Tehran University of Medical Sciences (TUMS), P.O. Box 14155-6117, Tehran, Iran; 3 Food Microbiology Research Center, Tehran University of Medical Sciences, Tehran, Iran

**Keywords:** Metabolic healthy, Metabolic unhealthy, Lifeline Diet Score, Obesity and overweight, Karelis criteria

## Abstract

**Background:**

Previous studies have shown the association of a number of dietary quality scores with metabolically phenotypes of obesity. Recently, the Lifelines Diet Score (LLDS), which is a fully food-based score based on the 2015 Dutch dietary guidelines and underlying international literature, has been proposed as a tool for assessing the quality of the diet. Therefore, this study was performed to investigate the association between LLDS and metabolically healthy/unhealthy overweight and obesity (MHO/MUHO) phenotypes.

**Methods:**

This study was performed on 217 women, aged 18–48 years old. For each participant anthropometric values, biochemical test and body composition were evaluated by standard protocols and methods. The LLDS was determined based on 12 components using a valid and reliable food frequency questionnaire (FFQ) containing 147 items. The metabolically healthy (MH) was evaluated using the Karelis criteria.

**Results:**

Among the total participants in this study, 31.3% of the subjects were MHO while 68.7% were MUHO. After adjustment for potential confounding variables (age, energy intake, and physical activity), participants in highest LLDS tertile had a lower odds of MUHO compared with those in the lowest tertile (OR: 1.18; 95% CI: 0.23, 5.83; *P*-trend = 0.03). Also, after further adjustment with BMI, provided only small changes in "OR" and did not attenuate the significance (OR: 1.28; 95% CI: 0.23, 6.91; *P*-trend = 0.02).

**Conclusions:**

The present evidence indicates that individuals with higher adherence to the LLDS had lower odds of metabolically unhealthy (MUH).

## Introduction

Obesity, as one of the most important metabolic diseases with phenotypic diversity, has a growing prevalence in all age groups around the world [1–3]. A subset of obesity that has recently attracted a great deal of attention is the metabolically healthy overweight/obesity (MHO), in which individuals, despite having excessive body fatness, show a favorable metabolic profile compared to their counterparts with similar body mass index (BMI) [4–7]. People with the MHO phenotype are characterized by high levels of insulin sensitivity, low levels of inflammatory markers, greater cardiorespiratory readiness, lower visceral fat ratio, lower liver fat, the absence of any dyslipidemia or hyperlipidemia, and lower prevalence of hypertension [8, 9]. It is estimated that the prevalence of this phenotype is about one third of obese people [4]. Evidence suggests that MHO is a temporary condition and individuals do not remain in this phenotype permanently [10, 11]. One study found that over a period of 5–10 years, nearly 30% of people with the MHO phenotype changed to an unhealthy metabolic status [12]. Also, in another study, in a 6-year follow-up, nearly 30% to 40% of people with the MHO phenotype changed to another phenotype, metabolically unhealthy overweight/obesity (MUHO) [13]. Unlike MHO people, MUHOs show an increased risk of type 2 diabetes (T2D), cardiovascular disease (CVD), and various cancers and are also associated with obesity-related metabolic disorders such as insulin resistance (IR) and dyslipidemia [14]. Factors such as age, sex, genetics and lifestyle factors may be associated with the transition from MHO to MUHO [15–18].

The importance of nutrition in the development of non-communicable diseases is well known. Studies have shown that the quality of diet as one of the determinants of obesity [19], when low, is associated with diseases such as diabetes mellitus (DM) and metabolic syndrome (MetS) [20, 21]. Numerous dietary scores have been developed around the world to measure adherence to dietary guidelines and dietary patterns [22–24]. The Lifelines Diet Score (LLDS) has been proposed as a measure of the relative quality of the diet [25]. This score was calculated based on the Dutch dietary guidelines for 2015, which are based entirely on scientific evidence and detailed studies on the relationship between foods and dietary patterns with chronic diseases [25, 26]. This dietary score consists of nine food groups that have positive effects on health, including vegetables, fruits, whole grain products, legumes and nuts, fish, oils and soft margarines, unsweetened dairy, coffee, and tea, and three other food groups of red and processed meat, butter and hard margarines, and sugar-sweetened beverages that have negative effects on health [25, 26]. Studies have shown that intakes a healthy diet including fruits, vegetables, whole grains and fish is inversely associated with diabetes, CVD and MetS [27, 28], and intakes processed meats and sugary drinks is associated with an increased risk [29, 30]. On the other hand, receiving a healthier diet has been shown to be positively associated with the MHO phenotype [31]. Therefore, in view of the above and the fact that to date no study has examined the association between LLDS and metabolic phenotypes of obesity, this study was conducted with the aim of whether LLDS increases healthy metabolic phenotypes.

## Methods and materials

A group of 217 women aged 18–48 years participated in current study. The target population was randomly selected from obese women who were referred to health centers affiliated to Tehran University of Medical Sciences (TUMS). All participants signed a written consent form. The medical research ethics committee of TUMS approved the study with the following identification. All anthropometric assessments and measurements were obtained by an experienced nutritionist. Participants had a BMI ≥ 25 kg/m^2^ to enter the study. Exclusion criteria include any acute or chronic illness, including cardiovascular disease, diabetes, cancer, thyroid disease, kidney disease, as well as people who are currently pregnant, breastfeeding, menopause, consuming alcohol and smoking, special diet and regular use of any medications or supplements. Also, people with energy intake < 800 kcal/d and 4200 kcal/d < were excluded from the study.

### Measurement of biochemical parameters

A complete description of the measurement method is given in the previous study [9]. Following an overnight fasting, fasting blood sugar (FBS), triglyceride (TG), total cholesterol, high-density lipoprotein (HDL), low density lipoprotein (LDL), and high-sensitive C-reactive protein (hs-CRP), serum insulin concentrations, were measured [9].

### Body composition and anthropometric measurements assessment

Body composition was assessed using the InBody 770 scanner. The protocol of which has been described in other studies [9]. Weight was measured using calibrated digital scales and height was measured by a wall-mounted stadiometer. Also, waist circumference (WC) from midpoint between the last rib and the iliac crest, and hip circumference (HC) the most prominent part is marked and without imposing any pressure on the body with a precision of 0.5 cm were calculated. BMI was also calculated as weight (kg) divided by height (m^2^).

### Assessment of blood pressure

To measure blood pressure, participants rested for 10–15 min, then blood pressure was measured using sphygmomanometer (Omron, Germany, European).

### The HOMA-IR calculation

The insulin resistance homeostatic model assessment (HOMA-IR) according to the following equation: [fasting plasma glucose (mmol/l) × fasting plasma insulin (mIU/l)]/22.5 [32].

### Definition of metabolic health and its components

We used karelis criteria according to which the presence of 4 or more of the following items indicates a healthy phenotype: TG ≤ 1.7 mmol/l, HDL ≥ 1.3 mmol/l and no treatment, LDL ≤ 2.6 mmol/l and no treatment, hs-CRP ≤ 3.0 mg/l, and HOMA-IR ≤ 2.7 [33].

### Assessment of dietary intake

A semi-quantitative food frequency questionnaire (FFQ) with 147 food items including a list of foods consumed during the past year was used to assess dietary intake. All participants were asked about the amount and frequency of each food item consumed daily, weekly or monthly. The reported frequency for each food item was then converted to grams/day. Evaluation of dietary nutrient intake was performed using N4 (First Data Bank, San Bruno, CA) software with its database adapted for Iranian foods. This questionnaire also had high validity and reliability [34].

### Lifelines Diet Score

The LLDS is a tool for ranking people about the relative quality of the diet, calculated using the method of Vinke et al. [25]. This dietary privilege is explained in detail elsewhere [25, 35]. In summary, according to the Dutch diet guidelines in 2015, which are completely based on scientific evidence, LLDS includes the consumption of nine food groups of vegetables, fruit, whole grain products, legumes and nuts, fish, oils and soft margarines, unsweetened dairy, coffee, and tea that have been proven to have positive effects on health, and three food groups including red and processed meat, butter and hard margarines, and sugar-sweetened beverages that have a negative effect on health. Individual’s food intake was expressed in grams per 1000 kcal. For each food group, intake was divided into 1 to 5 quintiles, that 5 points are given to the maximum intake and 1 point is given to the minimum intake of positive food groups, and for negative food groups, 5 points are awarded to the lowest intake and 1 point is given to the minimum intak. Finally, the sum of the scores of the 12 components is considered as LLDS score, which is in the range of 12–60 [25, 35].

### Assessment of other variables

To determine the level of physical activity of the participants, we used a validated international physical activity questionnaire (IPAQ). According to the IPAQ scoring protocol, individuals were divided into the following groups in terms of physical activity: (1) low active (< 600 MET-h/week); (2) moderate active (≥ 600 MET-h/week); and (3) high active (≥ 3000 MET-h/week) [36]. We also used a standard socio-demographic questionnaire to collect data age, education level, marital status, job, supplementation, and economic status.

### Statistical analysis

To evaluate the normal distribution of variables, the Kolmogorov–Smirnov test was used, which was normal. Data on continuous characteristics were reported as the mean ± standard deviations (SDs) and data on categorical characteristics were expressed as percentages and numbers. Chi-square test was used to evaluate significant differences of categorical variables among tertiles of LLDS score and one-way analysis of variance (ANOVA) was used to evaluate significant mean differences of continuous variables across tertiles of LLDS cutoff points (T1: ≤ 34, T2: 35–38, T3 ≥ 39). The post-hoc multiple comparison analysis by bonferroni has shown a significant mean difference between the groups. The analysis of covariance (ANCOVA) was used to identify dietary intakes and general characteristics mean differences between tertiles of the LLDS after adjusted by energy intake for the dietary intakes and further with age, physical activity, and BMI for general characteristics. We used binary logistic regressions to assess the association between LLDS score with obesity phenotypes and estimate odds ratios (ORs) and 95% confidence intervals (CIs) for the presence of MUHO with LLDS score in crude and multivariable-adjusted models. Age, energy intake, and physical activity were controlled for in the first model. Further adjustment was made for BMI in the second model. The first tertile of LLDS score and MHO were considered as the reference categories. In the present study *P* values < 0.05 and *P* values = 0.05, 0.06 and 0.07 were considered as significant levels and marginally significant, respectively. All statistical analyzes via statistical package for social sciences (version 24; SPSS Inc., Chicago, IL, USA) was performed.

## Result

### Study population characteristics

A total of 217 obese women, 31.3% of the subjects were classified as MHO and 68.7% were MUHO, according to Karelis criteria. At this study population the mean and standard deviation (SD) age, height, weight, and BMI of participants were 36.40 (8.40) year, 161.41 (5.85) cm, 80.30 (11.33) kg, and 30.80 (3.82) kg/m2 respectively. Moreover, the mean (SD) indicator of Karelis criterion including TG, HDL, LDL, hs-CRP, and HOMA-IR of participants were 1.32 (0.76), 1.14 (0.24), 2.39 (0.62), 4.26 (4.57), and 3.36 (1.33), respectively. Among the participants, 175 (76.8%) women were married and 95 (41.7%) had a moderate economic status.

### General characteristics of study population among tertiles of the LLDS score

The baseline characteristics of study participants, categorized according to the LLDS score, were presented in Table 1. As shown in this table, in the crude model, a significant mean difference was observed among tertiles of the LLDS score in terms of FFM (*P* = 0.04) and marginally significant for age (*P* = 0.06). After controlling for potentially confounding variables (age, energy intake, physical activity, BMI), the marginally significant was observed for hs-CRP (*P* = 0.06), which according to bonferroni post-hoc testing, the mean difference in hs-CRP was between T1 and T3, such that the mean was higher in T3. Also, after adjustment with energy intake, physical activity, BMI, there was a significant mean difference for the age of participants among tertiles of the LLDS score (*P* = 0.03), this mean difference was between T1 and T3 according to bonferroni post-hoc testing, so that age mean was more in T3 than T1. BMI was considered as collinear for anthropometrics and body composition variables. There were no significant mean differences in other variables among tertiles of LLDS scores (*P* > 0.05).

**Table 1:** General characteristics of study population among tertiles of the LLDS score in obese and overweight women (n = 217)

Variables	Tertiles of the LLDS score			*P* value	*P* value*
	T_1_ (n = 76) ≤ 34	T_2_ (n = 80) 35–38	T_3_ (n = 61) ≥ 39		
Quantitative variable	Mean ± SD				
*Demographic characteristic*					
Age (Y)	34.66 ± 8.66 ^a^	35.89 ± 8.36	38.08 ± 7.98 ^a^	**0.06**	**0.03**
PA (MET-min/week)	1046.03 ± 1953.25	1058.70 ± 1287.57	1389.63 ± 2207.04	0.50	0.45
*Anthropometry and Body Composition*					
Weight (kg)	78.81 ± 11.39	78.44 ± 9.10	81.03 ± 10.72	0.30	0.10
Height (cm)	162.27 ± 5.57	160.32 ± 5.57	161.29 ± 5.47	0.09	0.75
WC (cm)	95.22 ± 10.33	93.32 ± 15.81	96.22 ± 96.22	0.64	0.64
HC (cm)	112.93 ± 9.17	112.96 ± 6.90	114.29 ± 8.14	0.62	0.50
BMI (kg/)	29.91 ± 3.71	30.65 ± 3.47	30.98 ± 3.44	0.18	0.23
WHR	0.93 ± 0.05	0.92 ± 0.04	0.93 ± 0.05	0.81	0.41
BF (%)	40.75 ± 4.41	41.58 ± 5.08	40.94 ± 4.99	0.70	0.99
BFM (kg)	32.36 ± 7.39	32.94 ± 6.57	33.35 ± 7.37	0.16	0.62
FFM (kg)	46.36 ± 5.29	45.81 ± 5.35	47.48 ± 5.62	**0.04**	0.44
VFL	15.03 ± 3.22	15.43 ± 3.23	15.37 ± 3.33	0.69	0.43
FMI (kg)	12.30 ± 2.70	12.87 ± 2.75	13.01 ± 3.00	0.29	0.71
FFMI (kg)	17.60 ± 1.46	17.75 ± 1.50	18.19 ± 1.34	0.24	0.36
*Blood pressure*					
SBP (mmHg)	111.66 ± 11.67	110.92 ± 13.38	112.86 ± 15.23	0.70	0.38
DBP (mmHg)	78.02 ± 9.50	78.07 ± 11.04	78.36 ± 9.44	0.97	0.12
*Biochemical variables*					
Insulin (mIU/mL)	16.81 ± 8.03	15.51 ± 5.21	15.01 ± 5.02	0.22	0.37
TG (mmol/L)	1.19 ± 0.42	1.53 ± 1.12	1.43 ± 0.84	0.38	0.18
HDL-C (mmol/L)	1.10 ± 0.24	1.10 ± 0.23	1.09 ± 0.15	0.99	0.53
LDL-C (mmol/L)	2.38 ± 0.71	2.37 ± 0.62	2.21 ± 0.45	0.63	0.79
TC (mmo/l)	4.95 ± 1.14	4.45 ± 0.96	4.52 ± 0.84	0.20	0.52
HOMA index	3.40 ± 1.45	3.41 ± 1.30	3.14 ± 1.22	0.55	0.59
*Inflammatory parameter and other variables*					
hs-CRP (mg/L)	4.54 ± 4.87 ^a^	4.23 ± 4.47	3.98 ± 4.36 ^a^	0.77	**0.06**
Categorical variables*					
*Education*				0.41	0.40
Under diploma	9 (37.5)	7 (29.2)	8 (33.3)		
Diploma	19 (29.7)	22 (34.4)	23 (35.9)		
Above diploma	46 (36.2)	51 (40.2)	30 (23.6)		
*Marital status*				0.68	0.31
Single	17 (36.2)	19 (40.4)	11 (23.4)		
Married	57 (33.9)	61 (36.3)	50 (29.8)		
*Job*				0.54	0.13
non-employed	44 (32.4)	51 (37.5)	41 (30.1)		
Employed	29 (38.7)	28 (37.3)	18 (24.0)		
*Supplementation*				0.66	0.66
Yes	37 (32.2)	41 (35.7)	37 (32.2)		
No	29 (36.3)	30 (37.5)	21 (26.3)		
*Economic status*				0.22	0.19
Poor	14 (25.5)	23 (41.8)	18 (32.7)		
Moderate	32 (34.8)	39 (42.4)	21 (22.8)		
Good	24 (42.9)	16 (28.6)	16 (28.6)		

Values are represented as means ± SD

Chi-square was used for categorical variables

categorical variables: N (%)

*BF%* body fat percentage; *BFM* body fat mass; *BMI* body mass index; *DBP* diastolic blood pressure; *FFM* fat free mass; *FFMI* fat free mass index; *FMI* fat mass index; *HDL-C* high density lipoprotein cholesterol; *HC* hip circumference; *HOMA*; homeostatic model assessment; *hs-CRP* high-sensitivity C-reactive protein; *LDL-C* low density lipoprotein cholesterol; *LLDS* Lifelines Diet Score; *PA* physical activity; *SBP* systolic blood pressure; *SD* Standard Deviation; *T* tertile; *TC* total cholesterol; *TG* triglyceride; *VFL* visceral fat level; *WC* waist circumference; *WHR* waist-hip ratio

*P* value: ANOVA test was used

*P* value*: ANCOVA was performed to adjusted potential confounding factors (age, energy intake, Physical activity, BMI), BMI consider as collinear variable for anthropometrics and body composition variables

*P* values < 0.05 were considered as significant levels and *P* values = 0.05, 0.06 and 0.07 were considered as marginally significant

^a^The significant difference was seen between T_1_ and T_3_

### Dietary intake of study subjects across tertiles of the LLDS score

Dietary intakes of participants across tertiles of LLDS score were presented in Table 2. After adjustment with the energy intake, there have shown the participants in the highest tertile of LLDS score had significantly higher intakes of vegetables, legumes, and nuts, fish, unsweetened dairy (*P* < 0.001), fruits (*P* = 0.001), oils, and soft margarine (*P* = 0.009), coffee (*P* = 0.004), but significantly lower intakes of red and processed meat (*P* = 0.01), butter and hard margarine (*P* < 0.001), sugar-sweetened beverages (*P* = 0.005). Also, after adjusting for energy intake there was a significant mean difference in dietary intakes of macronutrients and micronutrients, which indicated that the group with the highest adherence with LLDS had significantly higher intakes of protein, eicosapentaenoic acid (EPA), docosahexaenoic acid (DHA), iron, magnesium, potassium, manganese, beta carotene, pantothenic acid, vitamin B6, biotin, folate (*P* < 0.001), total fiber, vitamin A, vitamin k, selenium (*P* = 0.01), zinc (*P* = 0.002), copper (*P* = 0.004), chromium (*P* = 0.04), vitamin C (*P* = 0.003), vitamin D, caffeine (*P* = 0.02), thiamin, riboflavin (*P* = 0.007), carbohydrates (*P* = 0.001), whereas had significantly lower intakes of total fat, saturated fatty acid (SFA), calcium (*P* < 0.001), monounsaturated fatty acid (MUFA), (*P* = 0.005), linolenic acid (*P* = 0.03).

**Table 2:** Dietary intakes of study subjects according to tertiles of the LLDS score in obese and overweight women (n = 217)

Variables	Tertiles of the LLDS score			*P* value	*P* value*
	T_1_ (n = 76) ≤ 34	T_2_ (n = 80) 35–38	T_3_ (n = 61) ≥ 39		
Food groups	Mean ± SD				
*Positive food groups*					
Vegetables (g/d)	357.31 ± 256.94	424.34 ± 256.34	572.62 ± 254.26	** < 0.001**	** < 0.001**
Fruits (g/d)	504.46 ± 332.35	567.62 ± 365.52	668.98 ± 425.40	**0.03**	**0.001**
Whole grain products	450.27 ± 202.69	388.78 ± 155.21	467.14 ± 306.79	0.08	0.29
Legumes and Nuts (g/d)	51.06 ± 30.16	61.53 ± 46.22	87.12 ± 53.87	** < 0.001**	** < 0.001**
Fish	9.37 ± 11.85	9.71 ± 7.77	17.30 ± 14.48	** < 0.001**	** < 0.001**
Oils and soft margarines	12.54 ± 13.44	17.90 ± 15.91	20.22 ± 17.47	**0.01**	**0.009**
Unsweetened dairy	270.81 ± 232.52	333.90 ± 215.08	442.59 ± 264.84	** < 0.001**	** < 0.001**
Coffee	10.21 ± 26.19	22.44 ± 48.35	39.35 ± 71.45	**0.004**	**0.004**
Tea	764.44 ± 595.17	620.52 ± 476.88	805.71 ± 548.39	0.09	0.20
*Negative food groups*					
Red and processed meat	37.59 ± 24.84	28.25 ± 26.01	25.62 ± 19.55	**0.008**	**0.01**
Butter and hard margarines	25.10 ± 26.43	7.99 ± 12.66	3.70 ± 7.81	** < 0.001**	** < 0.001**
Sugar-sweetened beverages	41.47 ± 48.41	29.10 ± 78.30	9.68 ± 15.36	**0.005**	**0.005**
*Macronutrients*					
Energy (kcal)	2725.86 ± 813.05	2497.91 ± 762.85	2674.12 ± 663.23	0.14	–
Carbohydrates (g/d)	374.39 ± 125.36	359.81 ± 119.95	394.51 ± 122.16	0.25	**0.001**
Total Fat (g/d)	105.34 ± 36.44	88.64 ± 32.95	88.50 ± 22.46	**0.001**	** < 0.001**
Protein (g/d)	87.15 ± 31.60	84.78 ± 25.82	98.08 ± 23.73	**0.01**	** < 0.001**
*Micronutrients*					
MUFA (g/d)	34.24 ± 12.54	29.80 ± 11.96	29.06 ± 7.05	**0.01**	**0.005**
PUFA (g/d)	21.52 ± 9.70	19.23 ± 9.52	18.90 ± 5.37	0.14	0.21
SFA (g/d)	32.43 ± 13.89	25.91 ± 9.28	25.83 ± 8.12	** < 0.001**	** < 0.001**
Trans fat	0.001 ± 0.002	0.001 ± 0.003	0.001 ± 0.002	0.91	0.93
Linolenic acid (g/d)	1.39 ± 0.67	1.17 ± 0.62	1.13 ± 0.54	**0.02**	**0.03**
Linoleic acid (g/d)	18.73 ± 9.27	16.62 ± 9.02	16.08 ± 5.10	0.12	0.18
EPA (g/d)	0.02 ± 0.03	0.02 ± 0.02	0.05 ± 0.04	** < 0.001**	** < 0.001**
DHA (g/d)	0.08 ± 0.11	0.08 ± 0.07	0.17 ± 0.14	** < 0.001**	** < 0.001**
Iron (mg/d)	18.71 ± 6.29	17.61 ± 5.87	20.38 ± 5.65	**0.02**	** < 0.001**
Zinc (mg/d)	13.12 ± 4.65	12.40 ± 4.13	14.25 ± 3.86	**0.03**	**0.002**
Selenium (mcg/d)	120.80 ± 42.82	110.48 ± 40.08	130.32 ± 40.34	**0.01**	**0.01**
Copper (mg/d)	1.89 ± 0.65	2.00 ± 0.86	2.21 ± 0.63	**0.03**	**0.004**
Calcium (mg/d)	1091.29 ± 464.15	1137.74 ± 369.64	1337.28 ± 406.98	**0.002**	** < 0.001**
Magnesium (mg/d)	441.32 ± 144.65	442.61 ± 151.78	527.85 ± 134.08	**0.001**	** < 0.001**
Potassium (mEq/d)	4011.97 ± 1584.91	4266.76 ± 1511.92	5074.40 ± 1478.39	** < 0.001**	** < 0.001**
Manganese (mg/d)	6.98 ± 2.49	6.51 ± 2.42	7.97 ± 2.45	**0.002**	** < 0.001**
Sodium (mg/d)	4288.95 ± 1588.14	4123.72 ± 1414.90	4341.27 ± 1266.01	0.63	0.77
Chromium (mg/d)	0.09 ± 0.08	0.10 ± 0.08	0.13 ± 0.08	**0.03**	**0.04**
Vitamin C (mg/d)	171.45 ± 106.54	205.11 ± 156.15	221.07 ± 119.66	**0.07**	**0.003**
Vitamin E (mg/d)	16.02 ± 8.74	17.68 ± 10.82	17.60 ± 6.23	0.44	0.14
Vitamin A (mg/d)	730.91 ± 425.67	780.92 ± 439.60	919.01 ± 385.97	**0.03**	**0.01**
Beta carotene (mg/d)	4248.99 ± 2626.88	5447.76 ± 4490.96	6742.44 ± 3344.36	** < 0.001**	** < 0.001**
Vitamin K (mg/d)	171.64 ± 101.04	219.08 ± 293.04	275.92 ± 176.99	**0.01**	**0.01**
Vitamin D (ug/d)	1.69 ± 1.62	2.06 ± 1.63	2.42 ± 1.65	**0.03**	**0.02**
Thiamin (mg/d)	2.08 ± 0.69	1.94 ± 0.57	2.21 ± 0.63	**0.04**	**0.007**
Riboflavin (mg/d)	2.14 ± 0.92	2.18 ± 0.86	2.41 ± 0.68	0.14	**0.007**
Niacin (mg/d)	25.42 ± 10.73	23.96 ± 8.13	26.63 ± 6.80	0.20	0.25
Pantothenic acid (mg/d)	6.216 ± 3.25	6.23 ± 1.86	7.42 ± 2.03	**0.007**	** < 0.001**
Vitamin B6 (mg/d)	2.05 ± 0.75	2.13 ± 0.70	2.42 ± 0.63	**0.008**	** < 0.001**
Biotin (mg/d)	34.64 ± 21.64	37.41 ± 14.35	46.61 ± 14.04	** < 0.001**	** < 0.001**
Folate (mcg/d)	583.90 ± 180.65	577.09 ± 156.93	676.42 ± 175.99	**0.001**	** < 0.001**
Vitamin B12 (mcg/d)	4.74 ± 3.22	4.26 ± 2.01	4.58 ± 2.17	0.49	0.94
Total fiber (g/d)	43.17 ± 21.09	44.13 ± 17.08	49.24 ± 17.01	0.14	**0.01**
Caffeine (mg/d)	161.13 ± 120.86	132.86 ± 94.19	163.67 ± 108.45	0.15	**0.02**

Values are represented as means ± SD

*DHA* Docosahexaenoic Acid; *EPA* Eicosapentaenoic Acid; *MUFA* monounsaturated fatty acid; *PUFA* polyunsaturated fatty acid; *SD* Standard Deviation; *SFA* saturated fatty acid; *T* tertile

*P* value: ANOVA test was used

*P* value*: ANCOVA was performed to adjusted potential confounding factors (energy intake)

*P* values < 0.05 were considered as significant levels and *P* values = 0.05, 0.06 and 0.07 were considered as marginally significant

### The association between LLDS score with obesity phenotypes

The association between LLDS score with obesity phenotypes and the OR and 95% CIs for MUHO across tertile categories of LLDS score compare to MHO as reference group is indicated in Table 3. There was no significant association between obesity phenotypes with LLDS score in the crude model (OR: 0.97; 95% CI: 0.24, 3.89; *P* = 0.97), even after adjustment for potential confounding variables including age, energy intake, and physical activity in model 1 (OR: 1.18; 95% CI: 0.23, 5.83; *P* = 0.83) and also an additional adjustment for BMI in model 2 (OR: 1.28; 95% CI: 0.23, 6.91; *P* = 0.77), no association was observed. However, the association between LLDS score and obesity phenotypes had statistically significant trends across LLDS tertiles. In the crude model, the odds of MUHO compared to MHO had higher with more adherence of LLDS, and there was not statistically significant trend (*P*-trend = 0.58), but after adjustment for potential confounding variables (age, energy intake, and physical activity) in model 1, participants in highest LLDS tertile had a lower odds of MUHO compared with those in the lowest tertile (OR: 1.18; 95% CI: 0.23, 5.83; *P*-trend = 0.03). Model 2, with further adjustment with BMI, provided only small changes in "OR" and did not attenuate the significance (OR: 1.28; 95% CI: 0.23, 6.91; *P*-trend = 0.02).

**Table 3:** Crude and multivariable-adjusted odds ratios and 95% CIs for obesity phenotypes (MHO in comparison to MUO) across tertiles of LLDS score in obese and overweight women (n = 217)

Models	Tertile of the LLDS score			*P* trend
	T_1_ (n = 76) ≤ 34	T_2_ (n = 80) 35–38	T_3_ (n = 61) ≥ 39	
	OR (95%CI)			
Crude	Ref	0.90 (0.23–3.52)	0.97 (0.24–3.89)	0.58
Model 1	Ref	1.34 (0.27–6.73)	1.18 (0.23–5.83)	**0.03**
Model 2	Ref	1.34 (0.20–6.49)	1.14 (0.21–5.88)	**0.02**

Metabolically healthy is a reference group

Model 1: Adjusted for age, energy intake, and physical activity

Model 2: Model 1 further adjustment with BMI

*CI* Confidence Interval; *OR* odds ratio; *T* tertile

*P* values are reported base on the Binary logistic regression test

*P* < 0.05 considered as significant

## Discussion

In the present study, we investigated the association between LLDS and healthy metabolic phenotype of obesity. This is the first study that investigats the association between LLDS and healthy metabolic phenotype of obesity. In this study, no association was found between LLDS and MH, but with increasing LLDS adherence, significant trends in MH odds were observed.

Our results showed that with increasing tertiles, the average age of individuals and positive food groups consumption, increased, and negative food groups consumption were decreased also the amount of hs-CRP decreased with increasing LLDS tertile. As mentioned in the introduction, the LLDS has been proposed as a measure of the relative quality of the diet based on the Dutch dietary guidelines [25], which with increasing LLDS adherence, the quality of the diet has increased. A study on women with MH and MUH found that hs-CRP levels decreased with increasing healthy eating pattern's tertile. Many studies have shown that in obese people, the level of inflammatory factors secreted by adipose tissue such as IL-6 and TNF-alpha increase, which stimulates the production and secretion of acute phase proteins in the liver [37]. Elevated plasma levels of these inflammatory factors and hs-CRP are associated with an increased risk of IR and CVD [38]. In healthy dietary pattern, intake of foods including vegetables, fruits are reported to be highly correlated with inflammatory markers, especially hs-CRP [9]. It has also been shown that a diet rich in vegetables, fruits, and low in total fat and saturated fats may reduce inflammatory markers [9]. The high content of vitamin C and fiber in fruits and vegetables may reduce hs-CRP [39]. In addition to the effect of fruits and vegetables on this inflammatory factor, intake of olives [40] and fish [41] is also effective in this healthy dietary pattern. Studies have shown that fish and seafoods are inversely associated with hs-CRP levels due to the presence of long-chain n-3 PUFAs [42].

A diet rich in vegetables, nuts, and fruits is associated with reducing inflammation due to the high contents of magnesium, fiber, and antioxidants [43]. As in the present study, with increasing LLDS adherence the level of hs-CRP decreased. Also, with increasing LLDS adherence, the amount of magnesium (Mg), potassium (K), and vitamin C has increased. High levels of Mg, phytochemicals, K, and vitamin C intake are associated with decreased IR, which is a factor in the development of MUH [44]. Previous studies have shown that high fruit and vegetable intake are associated with a lower risk of metabolic syndrome, CVD [28], and also stated that fruits intake is associated with a lower risk of type 2 diabetes [45, 46]. In the present study, more LLDS adherence is associated with higher calcium (Ca), Mg, total fiber, and lower total fat intake, which decreased hs-CRP levels [47, 48]. Consumption of butter and hard margarines, red and processed meats also decreased with the increaseing tertiles, as reported in previous data an unhealthy diet is associated with a higher chance of MUH, which includes consuming more solid fats, red meat, brain, liver and kidney are known [9]. The Dutch lifelines cohort study done by Slagter et al. on dietary patterns in people aged 30–69 years, found that in the upper quartile of a healthier diet, rich in vegetables, fruits, and fish, unsweetened fermented milk products, and the prohibition of sweet beverages and sweet snacks; have a higher chance of MH in women [31]. As in our study with increasing LLDS adherence significant trends in MH odds were observed. Epidemiological and experimental studies have also shown that dairy intake, has beneficial effects on metabolic syndrome risk factors, and associated with reducing risk of obesity, CVD and body fat gain [49]. As in our study, consumption of unsweetened fermented dairy products increased, and consumption of sugar-sweetened beverages decreased with increasing tertiles.

While carbohydrate intake in participants increased by increasing tertiles, fiber intake has also increased. In the Dutch cohort study, it was observed that the consumption of bread, potatoes and sweet snacks was inversely related to MH, which is due to the high content of carbohydrates, especially refined carbohydrates, and high glycemic index, and low fiber content [50]. Also, high glycemic index and low fiber content are associated with IR and impaired glucose tolerance, followed by metabolic syndrome [51], while increasing carbohydrates intakes was associated with increased fiber intake, which may be associated with an increased odds of MH. In general, studies and data showed that receiving a healthy diet that is rich in vitamins, minerals, phytochemicals and etc. is effective in a healthy metabolic obesity.

The strengths of this study are the use of the FFQ questionnaire validated in the Iranian population. Examining this association in one gender and only in Tehran is one of the weaknesses of this study because these results can not be attributed to the entire Iranian society and all individuals.

## Conclusion

The LLDS did not have a significant association with MH, but it was found that further adherence to the LLDS could have significant trends with odds of MH. So, it is recommended to increase adherence to LLDS and healthy dietary pattern in obese people to increase the odds of MH. More longitudinal studies are needed to confirm these findings through replication in more diverse populations and at last to confirm this correlation.

## Data Availability

The data that support the findings of this study are available from Khadijeh Mirzaei but restrictions apply to the availability of these data, which were used under license for the current study, and so are not publicly available. Data are however available from the authors upon reasonable request and with permission of Khadijeh Mirzaei.

## Abbreviations

BF%: Body fat percentage
BFM: Body fat mass
BMI: Body mass index
CIs: Confidence intervals
CVD: Cardiovascular diseases
DBP: Diastolic blood pressure
DHA: Docosahexaenoic Acid
EPA: Eicosapentaenoic Acid
FBS: Fasting blood sugar
FFM: Fat free mass
FFMI: Fat free mass index
FMI: Fat mass index
HC: Hip circumferences
HDL-C: High density lipoprotein cholesterol
hs-CRP: High-sensitivity C-reactive protein
IR: Insulin resistance
IPAQ: International physical activity questionnaire
LDL-C: Low density lipoprotein cholesterol
LLDS: Lifelines Diet Score
MH: Metabolically healthy
MHO: Metabolically healthy overweight/obesity
MUH: Metabolically unhealthy
MUHO: Metabolically unhealthy overweight/obesity
PA: Physical activity
SBP: Systolic blood pressure
SDs: Standard deviations
SFA: Saturated fatty acid
T: Tertile
T2D: Type 2 diabetes
TBW: Total body water
TC: Total cholesterol
TG: Triglyceride
WC: Waist circumference
WHR: Waist-hip ratio
